# The role of 5-aminolevulinic acid in spinal tumor surgery: a review

**DOI:** 10.1007/s11060-018-03080-0

**Published:** 2018-12-29

**Authors:** John V. Wainwright, Toshiki Endo, Jared B. Cooper, Teiji Tominaga, Meic H. Schmidt

**Affiliations:** 1 Department of Neurosurgery, Westchester Medical Center/New York Medical College, 100 Woods Road, Macy Suite 1332, Valhalla, NY 10595 USA; 2 Department of Neurosurgery, Graduate School of Medicine, Tohoku University, Sendai, Japan

**Keywords:** 5-Aminolevulinc acid, Spinal neoplasm, Fluorescence guided resection, Protoporphyrin IX

## Abstract

**Purpose:**

Primary intradural spinal neoplasms account for a small proportion of central nervous system tumors. The primary treatment for these tumors consists of maximal safe resection and preservation of neurologic function. Gross total resection, which is associated with the lowest rate of tumor recurrence and longer progression-free survival for most histologies, can be difficult to achieve. Currently, the use of 5-aminolevulinc acid (5-ALA) which takes advantage of Protoporphyrin IX (PpIX) fluorescence, is a well-established technique for improving resection of malignant cerebral gliomas. This technique is being increasingly applied to other cerebral neoplasms, and multiple studies have attempted to evaluate the utility of 5-ALA-aided resection of spinal neoplasms.

**Methods:**

The authors reviewed the existing literature on the use of 5-ALA and PpIX fluorescence as an aid to resection of primary and secondary spinal neoplasms by searching the PUBMED and EMBASE database for records up to March 2018. Data was abstracted from all studies describing spinal neurosurgical uses in the English language.

**Results:**

In the reviewed studies, the most useful fluorescence was observed in meningiomas, ependymomas, drop metastases from cerebral gliomas, and hemangiopericytomas of the spine, which is consistent with applications in cerebral neoplasms.

**Conclusions:**

The available literature is significantly limited by a lack of standardized methods for measurement and quantification of 5-ALA fluorescence. The results of the reviewed studies should guide future development of rational trial protocols for the use of 5-ALA guided resection in spinal neoplasms.

**Electronic supplementary material:**

The online version of this article (10.1007/s11060-018-03080-0) contains supplementary material, which is available to authorized users.

## Background

Primary intradural spinal neoplasms account for a small proportion of central nervous system (CNS) tumors. The combined incidence of malignant and non-malignant primary spinal tumors in the United States is only 0.97 per 100,000 persons [1]. In a review of the National Cancer Database, primary tumors of the spinal cord, meninges, or cauda equina represented just 4.5% of all patients diagnosed with primary tumors of the CNS [2]. The majority of these tumors are benign (48.6–62.4%); the remainder are either borderline but non-malignant (12.8–15.6%) or malignant (22-38.6%) [1, 2]. Histologically, meningiomas are the most common primary spinal tumors, followed by nerve sheath tumors, ependymomas, and astrocytomas [1–3]. Gross total resection is associated with the lowest rate of tumor recurrence and longer progression-free survival for most histologies. Currently, the primary treatment for these tumors consists of maximal safe resection with preservation of neurologic function [4–10]. Unfortunately, subtotal resection is not uncommon particularly with intramedullary ependymoma (19–44%) and astrocytoma (60–94%), in part because of premature termination of surgery for deterioration in intraoperative neurophysiological monitoring, residual tumor not visible with conventional surgical techniques, an indistinct plane of resection, or diffuse infiltration of the spinal cord [3–6, 9, 11–15].

Fluorescence-guided resection of malignant cerebral gliomas utilizing 5-aminolevulinic acid (5-ALA) and protoporphyrin IX (PpIX) accumulation in tumors has become a well-established technique to facilitate greater extent of resection resulting in improved progression free survival [16–23]. 5-ALA has also been used with varying success in other cerebral neoplasms, such as meningiomas, medulloblastomas, ependymomas, and carcinoma metastases [24–29]. The utility of 5-ALA guided resection of spinal neoplasms has not been determined, but there is a growing body of literature describing its use. The purpose of this review is to identify and summarize the published reports on the use of 5-ALA in spinal neoplasms and define the histopathologic entities that are potential candidates for 5-ALA guided resection.

## Methods

### Search strategy and data extraction

A literature search of the PUBMED and EMBASE databases was conducted using the keywords “5-aminolevulinic acid,” “spinal,” “spinal tumor,” and “fluorescence,”. See Supplement 1 for search strings. All identified records from January 1, 1964 to March 1, 2018 were reviewed. Records were included if they described the use of 5-ALA-aided resection for spinal neoplasms in humans. Records were excluded if they described the use of 5-ALA for resection of cranial tumors, for non-neurosurgical uses, or if a fluorophore other than 5-ALA was used. Publications written primarily in languages other than English were also excluded. No records were found to be duplicates.

Records were reviewed, and data were extracted regarding basic patient demographics, spinal segment of the tumor, tumor location (e.g. extradural [ED]; intradural, extramedullary [ID-EM]; intradural, intramedullary [IM]), histology, World Health Organization (WHO) Grade, and presence or absence of fluorescence and its characteristics. Because of the lack of standardization in quantifying fluorescence any reported degree of fluorescence was considered a positive result and any further characterization was noted. Data for individual patients were provided in all records.

## Results

### Study selection and patient characteristics

**Fig. 1:**
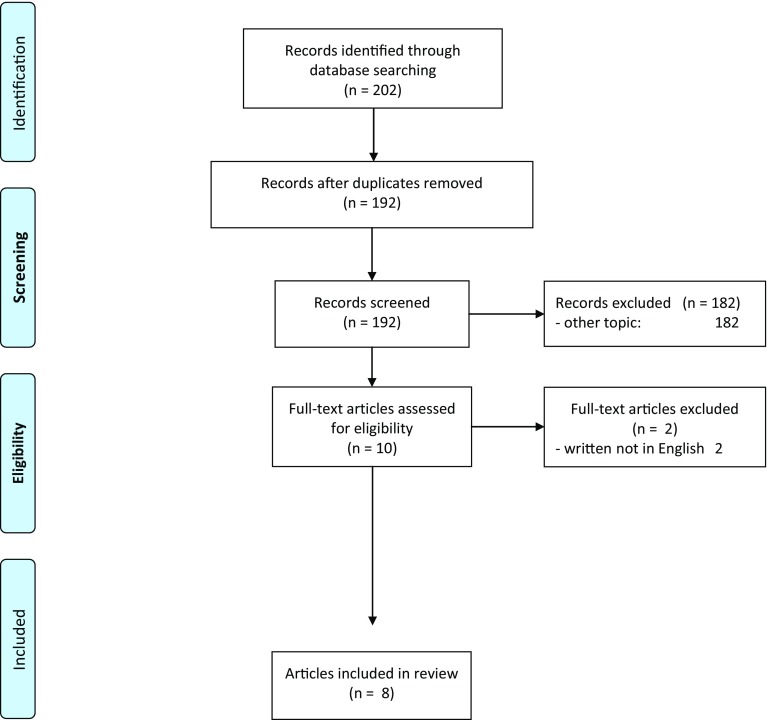
Flowchart showing literature search method

A total of 202 records were identified through our initial database search. De-duplication left 192 unique records. Titles and abstracts of these records were screened and 182 records were excluded as not relevant to the review yielding 10 records. Full texts of these records were reviewed and 2 articles written in non-English languages were excluded. The remaining 8 articles, which are the subject of this review, are summarized in Tables 1, 2 and 3 [3, 13, 22, 30–34]. A detailed flow chart of study identification and selection is presented in Fig. 1. For a full table of all abstracted data see Supplement 2. Screening of the references of included studies did not yield any additional articles for inclusion.

**Table 1:** List of included studies

Publication	Tumors included	ALA dose	Light source	Fluorescence positivity
Shimizu et al. [31]	1	1 gram	Laser device	1/1 (100%)
Ewelt et al. [32]	1	20 mg/kg BW	Violet-blue light microscope	1/1 (100%)
Rapp et al. [22]	2	Not reported	Not reported	2/2 (100%)
Inoue et al. [13]	10	20 mg/kg BW	Violet-blue light microscope	7/9 (77%)^a^
Muroi et al. [30]	1	20 mg/kg BW	Violet-blue light microscope	1/1 (100%)
Eicker et al. [33]	26	20 mg/kg BW	Violet-blue light microscope	12/26 (46%)
Millesi et al. [3]	55	20 mg/kg BW	Violet-blue light microscope	29/55
Krause Molle et al. [34]	1	20 mg/kg BW	Violet-blue light microscope	1/1 (100%)

^a^1 patient not administered 5-ALA

**Table 2:** List of included studies with histology

Publication	Tumors included	Histology (grade)	Fluorescence positivity
Shimizu et al. [31]	1	Ependymoma (WHO II) (n = 1)	1/1 (100%)
Ewelt et al. [32]	1	Anaplastic astrocytoma (WHO III) (n = 1)	1/1 (100%)
Rapp et al. [22]	2	Epidural metastasis (Anaplastic oligoastrocytoma) (n = 1)	1/1 (100%)
		Drop metastasis (Glioblastoma) (n = 1)	1/1 (100%)
Inoue et al. [13]	10	Ependymoma (WHO II) (n = 9)	6/8 (75%)^a^
		Anaplastic ependymoma (WHO III) (n = 1)	1/1 (100%)
Muroi et al. [30]	1	Meningothelial meningoma (WHO I) (n = 1)	1/1 (100%)
Eicker et al. [33]	26	Meningioma (n = 8) Meningothelial (WHO 1) (n = 6) Psammomatous (WHO I) (n = 1) Transitional (WHO I) (n = 1)	7/8 (75%) 6/6 (100%) 0/1 (0%) 1/1 (100%)
		Neurinoma/neurofibroma (n = 7) Neurinoma (WHO I) (n = 1) Neurofibroma (WHO I) (n = 1)	0/7 (0%) 0/1 (0%) 0/1 (0%)
		Gliomas (n = 4) Anaplastic astrocytoma (WHO III) (n = 1)^b^ Glioblastoma (WHO IV) (n = 1) Drop metastasis (Glioblastoma) (n = 1)^c^ Epidural metastasis (Anaplastic Astrocytoma) (n = 1)^c^	4/4 (100%) 1/1 (100%) 1/1 (100%) 1/1 (100%) 1/1 (100%)
		Other histologies (n = 7) Myxopapillary ependymoma (WHO I) (n = 2) Medulloblastoma (WHO IV) (n = 1) Metastatic choroid plexus papilloma (WHO I) (n = 1) Epidermoid cyst (n = 1) Intramedullary lipoma (n = 1) Demyelinating disease (n = 1)	1/7 (14%) 1/2 (50%) 0/1 (0%) 0/1 (0%) 0/1 (0%) 0/1 (0%) 0/1 (0%)
Millesi et al. [3]	55	Meningioma (n = 12) Meningothelial (WHO I) (n = 7) Transitional (WHO I) (n = 1) Psammomatous (WHO I) (n = 3) NOS (WHO I) (n = 1)	12/12 (100%) 7/7 (100%) 1/1 (100%) 3/3 (100%) 1/1 (100%)
		Hemangiopericytoma (WHO II) (n = 2)	2/2 (100%)
		Anaplastic Hemangiopericytoma (WHO III) (n = 1)	1/1 (100%)
		Chordoma (n = 2)	0/2 (0%)
		Ependymoma (n = 11) Ependymoma (WHO II) (n = 10) Anaplastic (WHO III) (n = 1)	11/11 (100%) 10/10 (100%) 1/1 (100%)
		Myxopapillary ependymoma (WHO I) (n = 1)	1/1 (100%)
		Neurinoma (WHO I) (n = 8)	0/8 (0%)
		Glioma (n = 3) Pilocytic astrocytoma (WHO I) (n = 1) Diffuse astrocytoma (WHO II) (n = 1) Anaplastic mixed oligoastrocytoma (WHO III) (n = 1)	0/3 (0%) 0/1 (0%) 0/1 (0%) 0/1 (0%)
		Hemangioblastoma (n = 2)	0/2 (0%)
		Paraganglioma (n = 1)	0/1 (0%)
		Ganglioglioma (n = 1)	0/1 (0%)
		Amelanotic melanocytoma (n = 1)	0/1 (0%)
		Metastasis (n = 7)	2/7 (28%)
		Giant cell tumor of bone (n = 1)	0/1 (0%)
		Lipoma (n = 1)	0/1 (0%)
		Malignant peripheral nerve sheath tumor (n = 1)	0/1 (0%)
Krause Molle et al. [34]	1	Leptomeningeal Spread of Anaplastic Astrocytoma (n = 1)	1/1 (100%)

^a^1 patient not administered 5-ALA

^b^Previously reported in Ewelt et al. [32]

^c^Previously reported in Rapp et al. [22]

**Table 3:** List of histologies and fluorescence results

Histology	Fluorescence positivity
Meningioma (n = 21)	20/21 (95%)
Meningothelial (WHO I) (n = 14)	14/14 (100%)
Psammomatous (WHO I) (n = 4)	3/4 (75%)
Transitional (WHO I) (n = 2)	2/2 (100%)
NOS (WHO I) (n = 1)	1/1 (100%)
Hemangiopericytoma (n = 3)	3/3 (100%)
Hemangiopericytoma (WHO II) (n = 2)	2/2 (100%)
Anaplastic hemangiopericytoma (n = 1)	1/1 (100%)
Ependymoma (n = 22)	19/21^a^
Ependymoma (WHO II) (n = 20)	17/19 (89%)
Anaplastic ependymoma (WHO III) (n = 2)	2/2 (100%)
Myxopapillary ependymoma (n = 3)	2/3 (66%)
Glioma (n = 7)	7/7 (100%)
Pilocytic astrocytoma (n = 1)	0/1 (0%)
Diffuse astrocytoma (n = 1)	0/1 (0%)
Anaplastic astrocytoma (WHO III) (n = 3)	3/3 (100%)
Anaplastic oligoastrocytoma (WHO III) (n = 1)	1/1 (100%)
Glioblastoma (n = 1)	1/1 (100%)
Neurinoma (n = 15)	0/15 (0%)
Metastases (n = 12)	6/12 (50%)
Medulloblastoma (n = 1)	0/1 (0%)
Chordoma (n = 2)	0/2 (0%)
Other histologies (n = 11)	0/11 (0%)

^a^1 of the 22 patients with ependymoma did not receive 5-ALA

There were 5 case reports and 3 prospective case series in the 10 articles reviewed. The literature described 91 patients (53 female, 44 male, average age 44.5 years) with 97 tumors; when duplicate patients are excluded, there were 94 unique tumors [3, 13, 22, 30–34]. In the reports where 5-ALA dosing information was provided, patients received 20 mg/kg orally 2–3 h prior to induction of anesthesia in all cases except those reported by Shimizu et al. [31], who administered 1 gm of 5-ALA orally 2 h prior to induction. Fluorescence was usually visualized using a neurosurgical microscope with integrated 5-ALA-PpIX fluorescence visualization capability that allows for switching between white light and blue excitation light for fluorescence visualization; Shimizu et al. [31] used an external semiconductor laser excitation source. ID-EM tumors made up the majority of tumors (n = 52, 55.3%), followed by IM (n = 36, 38.3%), and ED (n = 6, 6.4%) tumors. Meningiomas (n = 21, 22.34%) were the most common histology followed by intramedullary ependymoma (n = 22 23.41%), neurinoma (n = 14, 14.89%), drop metastases from primary cerebral tumors (n = 6, 6.38%), and epidural metastases (n = 5, 5.32%). All other tumor histologies individually represented less than 5% of the reported cases. No studies reported significant 5-ALA-related complications. The results of the studies are summarized by histology.

### Meningioma

Three studies reported the use of 5-ALA and PpIX fluorescence in the resection of 21 meningiomas [3, 30, 33]. All meningiomas resected were WHO grade I. Meningothelial meningiomas represented the most frequent histology (n = 11, 52.38%), followed by transitional (n = 4, 19.05%) and psammomatous (n = 4, 19.05%) meningiomas, metaplastic (n = 1, 4.76%), and 1 case reported as meningioma NOS (4.76%). Of the cases reported, all but one (95%) demonstrated positive PpIX fluorescence.

Muroi et al. [30] published the first report of the use of 5-ALA in the resection of a spinal meningioma in 2012. The authors describe the 5-ALA fluorescence-guided resection of a meningothelial meningioma in the cervical spine. Upon exposure of the tumor, they noted positive tumor fluorescence, but their report did not describe the fluorescence characteristics. Intermittent fluorescence visualization was used to detect tumor tissue, infiltration, and residual tumor during the resection. An apparent Simpson grade II resection of the tumor was achieved under white light visualization; however, under fluorescence visualization, residual tumor near the site of dural attachment was identified and resected. This fluorescence-positive remnant demonstrated identical histology to the bulk of the tumor. In this case, 5-ALA fluorescence selectively marked tumor tissue, enabling a more complete resection, and the patient was recurrence free on short-term, 1-year follow-up imaging [30].

Eicker et al. [33] a published a series of 26 spinal intradural tumors in 2013, which included 8 meningiomas. Fluorescence-guided resection revealed 3 meningothelial, 3 transitional, 1 psammomatous, and 1 metaplastic tumors; two tumors (1 meningothelial and 1 transitional) were recurrences. All tumors where WHO grade I. All tumors, except the psammomatous tumor, demonstrated positive fluorescence. This non-fluorescence was attributed to a delay (approximately 7 h) between the administration of 5-ALA and surgery. Alternatively, differences in ferrochelatase activity in meningioma cell lines that can reduce the accumulation of PpIX may have been responsible for the lack of fluorescence visualization [35]. Importantly, in the two cases of recurrent tumors, the authors reported that fluorescence was useful in distinguishing between areas of tumor and scar, with no fluorescence-negative tissue samples positive for tumor. In addition, they identified residual fluorescent tumor after apparent complete resection under white light in these same cases [33].

Millesi et al. [3] published the largest series of spinal tumors in which 5-ALA fluorescence was used. After presumed gross total tumor resection under white-light microscopy, a final inspection of the surgical cavity was performed to assess for residual fluorescence. In their series of 55 tumors, they reported 12 meningiomas (7 meningothelial, 3 psammomatous, 1 transitional, and 1 meningioma NOS), all WHO grade I. All tumors exhibited strong fluorescence; 10 (83.33%) tumors demonstrated homogeneous fluorescence and 2 (1 meningothelial and 1 psammomatous) demonstrated inhomogeneous fluorescence [3]. Using a MIB-1 antibody kit to evaluate proliferation rate in fluorescence-negative and -positive samples from the inhomogeneously fluorescing tumors, Millesi et al. observed a higher mean proliferation rate in the fluorescence positive areas which is consistent with data from cerebral gliomas [3, 13, 36, 37].

Together, these authors demonstrate the utility of 5-ALA fluorescence in the resection of spinal meningiomas. All spinal meningiomas in these studies were reported to have a gross total resection or a resection of at least Simpson grade II with 5-ALA fluorescence facilitating more complete resection. These data are in agreement with recent publications describing the use of 5-ALA in intracranial meningiomas [24, 29]. The ability to visualize residual tumor not identifiable under white light visualization is extremely beneficial as Simpson grade I-III resections are associated with symptom resolution and low recurrence rates [7, 8, 10, 29, 30, 33, 38]. Additionally, this ability to visualize tumor may allow for less aggressive surgery; the ability to target fluorescing tumor and visualize tumor as it arises from the dura, including any potential dural invasion, could facilitate more targeted resection to achieve a Simpson grade I-III result. However, this must be balanced with the potential for intratumoral heterogeneity and areas of non-fluorescing tumor that may mislead the surgeon.

### Ependymoma

Four studies reported the use of 5-ALA and PpIX fluorescence in the resection of 24 ependymomas [3, 13, 31, 33]. Intramedullary ependymomas represented the majority of the reported tumors (n = 22), of which 20 (91%) were ependymoma (WHO grade II) and 2 (9%) were anaplastic ependymoma (WHO grade III). Three myxopapillary ependymomas (WHO grade I) were reported as well. Eighty-seven percent of tumors exhibited positive fluorescence, with 2/2 (100%) of anaplastic intramedullary ependymomas (WHO grade III), 17/19 (89%) of intramedullay ependymomas (WHO grade II), and 2/3 (67%) of myxopapillary ependymomas (WHO grade I) exhibiting PpIX positivity [3, 13, 31, 33]. One patient with in Inoue et al.’s study did not receive 5-ALA.

Shimizu et al. [31] reported the first use of 5-ALA in the resection of a cervical intramedullary ependymoma (WHO I) in 2006. The tumor exhibited strong fluorescence, aiding in en bloc resection of the tumor. They achieved an apparent gross total resection under white light visualization and under conventional fluorescence visualization no residual tumor was visible. Further analysis of the fluorescence spectrum of the resection bed revealed peaks consistent with PpIX at the site of tumor attachment to the anterior raphe, suggesting residual foci of tumor. They proceeded with additional resection and final fluorescence examination of the tumor bed demonstrated no emission peaks, confirming complete removal of the tumor [31].

Inoue et al. [13] published the first series of intramedullary ependymomas resected utilizing 5-ALA fluorescence-guided resection in 2012. They reported 10 consecutive patients with intramedullary ependymoma who underwent resection from June 2006 to December 2010. One patient enrolled in the study refused to receive 5-ALA. Fluorescence-guided resection was performed, and histological analysis revealed 9 ependymomas (WHO grade II) and 1 anaplastic ependymoma (WHO grade III). Of the 9 patients who received 5-ALA, 7 (77%) demonstrated strong positive tumor fluorescence. Of the fluorescence-positive tumors, 4/7 (57%) exhibited uniform fluorescence and 3/7 (43%) demonstrated heterogenous fluorescence. The authors achieved gross total resection in 8 of 10 (80%) of patients. All of the patients who demonstrated positive fluorescence had complete resection of fluorescing tissue and demonstrated complete resection of tumor on follow-up; of the two fluorescence-negative tumors and the tumor in the patient who did not receive 5-ALA, total resection was accomplished in only one case. Inoue et al. [13] reported that 5-ALA fluorescence enabled more clear visualization of dissection planes and was particularly useful in delineating the ventral, cranial, and caudal margins of the tumors. All tumor samples taken from fluorescence-positive areas contained tumor. Additionally, cell proliferation indices were assessed using the MIB-1 antibody and demonstrated significantly higher proliferation indices for fluorescence-positive areas of tumor compared with negative areas [13].

Eicker et al. [33] reported on the resection of 2 myxopapillary ependymomas (WHO grade I). Only 1 of 2 ependymomas exhibited positive fluorescence, and the authors made no comment on the character of the fluorescence. They noted that, when present, fluorescence facilitated complete resection [33].

Millesi et al. [3] reported on the resection of 12 ependymomas. Ten were intramedullary ependymoma (WHO grade II), 1 was intramedullary anaplastic ependymoma (WHO grade III), and 1 was myxopapillary ependymoma (WHO grade I). For resections of these tumors, the authors utilized principles of fluorescence-guided resection to facilitate more complete removal of tumor. They observed strong positive fluorescence in 10/12 (83%) and weak fluorescence in 1 ependymoma (WHO grade II). Fluorescence was homogeneous in 5/12 (42%) of tumors. Similar to the previous reports, the authors were able to identify residual fluorescing foci in 4 intramedullary ependymomas that were positive for tumor upon resection. Gross total resection was achieved in 9 of 12 (75%) ependymomas. An infiltrative growth pattern, absence of a clear cleavage plane, and neuromonitoring changes were cited as the primary reasons for incomplete resection in their series overall [3].

As with the reported cases of spinal meningioma resected utilizing 5-ALA fluorescence, these reports demonstrate that 5-ALA fluorescence-guided resection of spinal ependymomas can facilitate more complete resection when the tumor exhibits positive fluorescence. When extent of resection was reported, gross total resection was achieved in 17/22 (77%) of cases and subtotal resection was reported in 5/22 (23%) of cases which is similar to prior reports [3, 13, 39, 40]. Given that extent of resection, along with histology, is the most important prognostic factor in post-surgical outcome and progression-free survival, 5-ALA guided resection of spinal ependymomas is a reliable and clinically significant adjunct to surgery in these patients [14, 38, 39, 41–43].

### Drop metastases of cerebral neoplasms

Three studies reported the use of 5-ALA fluorescence in the resection of 6 drop metastases from cerebral neoplasms [3, 22, 33]. Three lesions were drop metastases of cerebral glioblastomas (WHO grade IV), 1 was a medulloblastoma (WHO grade IV) metastasis, 1 was a choroid plexus papilloma metastasis (WHO grade I), and 1 was pineal gland papillary tumor (WHO grade II–III). All of the glioblastoma metastases and the pineal gland papillary tumor metastasis exhibited positive fluorescence, which the authors found it useful in aiding their resection of these tumors because it distinguished between tumor and normal tissue well. The medulloblastoma metastasis and the choroid plexus papilloma metastasis did not demonstrate fluorescence. Although, reports of spinal drop metastases of malignant cerebral gliomas are rare, the significant progress made in survival times can be assumed to lead to an increased frequency of these metastases [44, 45]. 5-ALA guided resection has proven to be beneficial in cerebral malignant gliomas and appears to be a feasible approach in spinal metastases, but it remains unclear from the available data whether this approach will provide clinical benefit to these patients. There is also insufficient data to determine whether 5-ALA-guided resection of other histologic types of drop metastases will prove beneficial, although data from cerebral medulloblastoma suggests varying fluorescence in this entity may limit the utility [25, 28].

### Other histologies with positive fluorescence

A very small number of other histologies were reported to have positive fluorescence, including 3 hemangiopericytomas (2 WHO grade II and 1 anaplastic WHO grade III), 1 intramedullary anaplastic astrocytoma (WHO grade III), 1 leptomeningeal metastasis of intramedullary anaplastic astrocytoma (WHO grade III), 1 ganglioglioma (WHO grade I), and 1 epidural metastasis of a cerebral anaplastic oligoastrocytoma (WHO grade III) [3, 22, 32, 34]. 5-ALA fluorescence may be useful in the resection of these entities although more data are needed. The application of 5-ALA in intramedullary astrocytoma is not likely to provide significant benefit despite the evidence that some tumors may exhibit positive fluorescence. In contrast to cerebral astrocytoma, exquisitely eloquent spinal cord tissue very intimately surrounds intramedullary tumors and is infiltrated by tumor cells, increasing the risk associated with resecting fluorescent tissue that may contain functional, normal tissue. Additionally, an increased extent of resection has not been shown to provide significant benefit to these patients [9].

### Fluorescence-negative tumors

In contrast to the above, none of the other histologies—which included 14 neurinomas, 5 epidural carcinoma metastases, 3 primary spinal gliomas, and 1 intramedullary carcinoma metastasis exhibited positive fluorescence (see Table 1 for the full list) [3, 33]. Based on the available data, 5-ALA does not appear to be useful for these tumors.

## Illustrative case

**Fig. 2:**
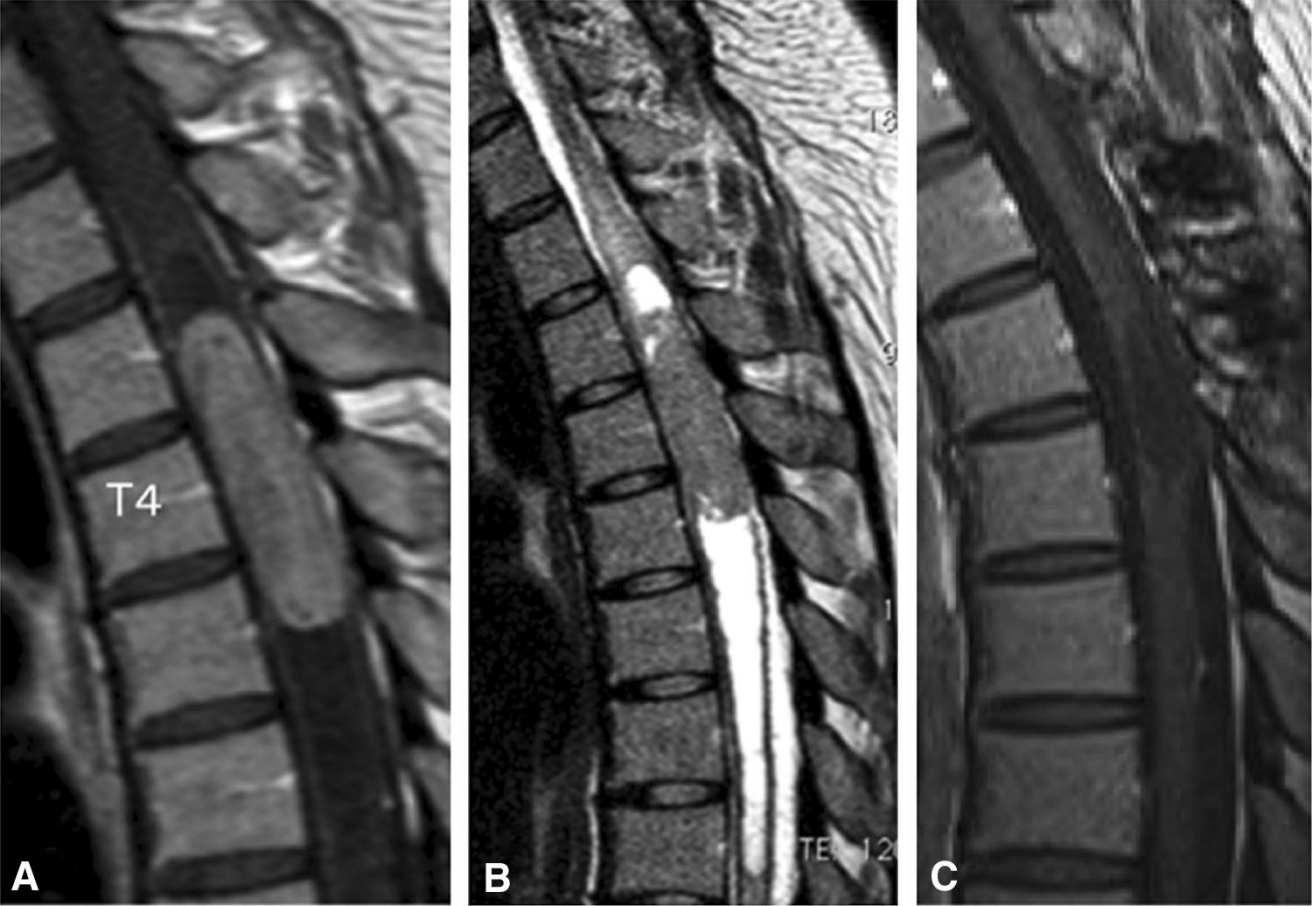
Magnetic resonance imaging (MRI) of the case described by Inoue et al. Preoperative T1-weighted (**a**) and T2 weighted (**b**) sagittal MRI revealed a homogeneously enhanced intramedullary T3-5 tumor with cyst formation extending to T8. Postoperative T1-weighted sagittal MRI (**c**) confirmed complete tumor resection with no signs of recurrence 2 years after surgery. Reprinted from Inoue et al. [13] 5-aminolevulinic acid fluorescence-guided resection of intramedullary ependymoma: Report of 9 cases. Neurosurgery 72:159–168. Reprinted with permission Oxford University Press

**Fig. 3:**
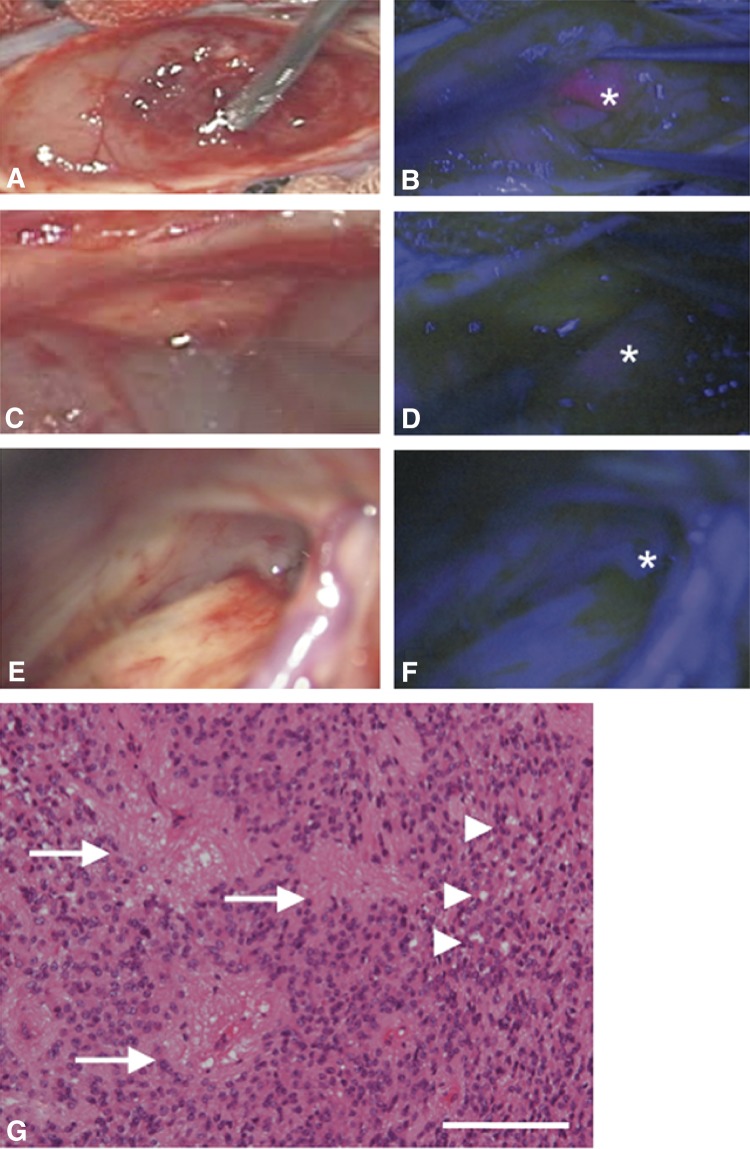
Intraoperative images of ependymoma resection described by Inoue et al. Note that the images obtained using white light (left column) are coupled with images using 5-aminolevulinic acid (5-ALA) fluorescence (right). (**a** and **b**) After dissection of the posterior medial sulcus, the tumor, which had a vivid reddish fluorescence (asterisk), was encountered. **c** Dissection of the lateral wall of the tumor from the spinal cord. The tumor was encapsulated and easily distinguished from the surrounding spinal cord parenchyma under the microscope. **d** A slight fluorescence was noted on the surface of the tumor (asterisk). **e** and **f** At the ventrocaudal edge of the tumor, gray tissue was left untouched on the basis of negative 5-ALA fluorescence (asterisk). **g** Characteristic perivascular pseudorosettes (arrows) and ependymal rosettes (arrowheads) were observed in the tissue with 5-ALA-positive fluorescence. Hematoxylin and eosin stain. Scale bars = 200 µm. Reprinted from Inoue et al. [13] 5-aminolevulinic acid fluorescence-guided resection of intramedullary ependymoma: Report of 9 cases. Neurosurgery 72:159–168. Reprinted with permission from Oxford University Press

Inoue et al. [13] described a 25-year-old woman who presented with a 3-month history of back pain and difficulty walking (McCormick grade III). Magnetic resonance imaging revealed a homogeneously enhancing intramedullary T3-5 tumor with central canal expansion observed from T6 to T8 (Fig. 2). After a discussion of the risks and benefits of surgical intervention, the decision was made to perform surgical resection of the lesion. 5-ALA was administered 2 h prior to induction, and the tumor was exposed through a standard, posterior midline approach. Excitation light demonstrated vivid, heterogenous red fluorescence of the tumor (Fig. 3). The tumor was resected; although the tumor appeared to be contiguous with the cyst wall at the cranial, caudal, and ventral aspects of the cyst wall however no fluorescence was identified and this tissue was not resected (Fig. 3). Histopathological analysis of fluorescence-positive tissue demonstrated ependymoma (WHO grade II) (Fig. 3). Samples from the cranial and caudal end of the resection bed, which were fluorescence-negative, were negative for tumor cells. Post-operative magnetic resonance imaging confirmed complete resection, the patient demonstrated improvement in her neurologic status, and there was no evidence of recurrence at 24.1 months postoperatively. This case highlights the utility of 5-ALA fluorescence in guiding tumor resection and reducing unnecessary resection of normal tissue which could lead to neurologic deficit [13].

## Discussion

Our review of the available literature describing the use of 5-ALA fluorescence during resection of spinal neoplasms found that positive fluorescence was observed most consistently and proved to be most beneficial with meningiomas, ependymomas, drop metastases from malignant cerebral gliomas, and hemangiopericytomas, consistent with what is observed in applications of 5-ALA in cerebral neoplasms [24, 29, 46].

A major limitation of the available literature is that a standardized method for the measurement and quantification of 5-ALA fluorescence has not been established. Because of this, most studies relied on subjective characterization which can introduce significant bias. No study was able to report on the sensitivity or specificity of fluorescence further limiting the conclusions that can be drawn from available data. Despite this, the theoretical and demonstrated advantages of enhanced tumor visualization make this technique a valuable adjunct to resection, provided the tumor exhibits positive fluorescence. The heterogeneity of the visible fluorescence response within these tumor entities can potentially reduce the utility of 5-ALA-guided resection; however, as has been demonstrated, 5-ALA-induced PpIX fluorescence of tumor tissue can be detected quantitatively [13, 24, 30, 31, 47, 48]. Real-time, quantitative measurement of PpIX fluorescence and other novel techniques such as confocal endomicroscopy during resection of tumors that do not homogenously fluoresce may be able to overcome this limitation [49].

Another limitation of the available reports is the lack of a standardized method of evaluating the preoperative imaging for characteristics that could predict useful fluorescence during surgery. Future studies should aim to predict whether 5-ALA fluorescence will be useful by assessing preoperative imaging so unnecessary administration of 5-ALA can be avoided. From the above reports, it is likely that suspected neurinomas are poor candidates for the use of 5-ALA.

Lastly, long-term follow-up is missing on the vast majority of patients reported in the literature. Until longer follow-up has been achieved and reported, data on the overall and progression-free survival rates remain incomplete and the benefits of 5-ALA-guided resection uncertain.

## Conclusion

In this review, we summarized the available literature describing the use of 5-ALA fluorescence during resection of spinal neoplasms, all of which is Level IV evidence. Positive fluorescence was observed most consistently and proved to be most beneficial to facilitate resection with meningiomas, ependymomas, drop metastases from malignant cerebral gliomas, and hemangiopericytomas, consistent with what is observed in intracranial applications of 5-ALA. These results should guide future development of rational trial protocols for the use of 5-ALA-guided resection of spinal neoplasms.

## Electronic supplementary material

Below is the link to the electronic supplementary material.

Supplementary material 1 (DOCX 14 KB)

Supplementary material 2 (DOC 237 KB)
