# Supplementary material for: The role of 5-aminolevulinic acid in spinal tumor surgery: a review
Source: J Neurooncol. 2018 Dec 29;141(3):575–84. doi: 10.1007/s11060-018-03080-0 (PMC6373300; doi:10.1007/s11060-018-03080-0)
Supplement: Supplementary file 1 — Supplementary material 1 (DOCX 14 KB) [file 11060_2018_3080_MOESM1_ESM.docx]

| **Supplement 1:** Listing of search terms. | | | |
| --- | --- | --- | --- |
| **PUBMED:** | | | |
| 1. (("fluorescence"[MeSH Terms] OR "fluorescence"[All Fields]) AND spin[All Fields] AND ("tumour"[All Fields] OR "neoplasms"[MeSH Terms] OR "neoplasms"[All Fields] OR "tumor"[All Fields])) AND ("1964/01/01"[PDAT] : "2018/03/01"[PDAT])   Results: 138   1. (("aminolevulinic acid"[MeSH Terms] OR ("aminolevulinic"[All Fields] AND "acid"[All Fields]) OR "aminolevulinic acid"[All Fields] OR "5 ala"[All Fields]) AND spinal[All Fields]) AND ("1964/01/01"[PDAT] : "2018/03/01"[PDAT])   Results: 32 | | | |
|  | | | |
| **EMBASE:** | | | |
| No. | Query | Results | Date |
| #7  #6  #5  #4  #3  #2  #1 | #3 AND #6  #4 OR #5  'spinal tumor'/exp OR 'spinal tumor' OR (spinal AND ('tumor'/exp OR tumor))  'spine tumor'/exp  #1 OR #2  '5-aminolevulinic acid'/exp OR '5-aminolevulinic acid' OR ('5 aminolevulinic' AND ('acid'/exp OR acid))  'aminolevulinic acid'/exp | 32  62,633  62,633  8,654  8,668  8,668  8,171 | 01 Mar 2018  01 Mar 2018  01 Mar 2018  01 Mar 2018  01 Mar 2018  01 Mar 2018  01 Mar 2018 |
