# Supplementary material for: The role of 5-aminolevulinic acid in spinal tumor surgery: a review
Source: J Neurooncol. 2018 Dec 29;141(3):575–84. doi: 10.1007/s11060-018-03080-0 (PMC6373300; doi:10.1007/s11060-018-03080-0)
Supplement: Supplementary file 2 — Supplementary material 2 (DOC 237 KB) [file 11060_2018_3080_MOESM2_ESM.doc]

| **Supplement 2.** Data from the studies included in this review. | | | | | | | | | | | | | |
| --- | --- | --- | --- | --- | --- | --- | --- | --- | --- | --- | --- | --- | --- |
| **Publication** | **Patient** | **Tumor** | **Case in Series** | **Age** | **Sex** | **Seg** | **Loc** | **Histology** | **WHO Grade** | **Fluorescence** | **Fluorescence Strength** | **Fluorescence Homogeneity** | **EOR** |
| **Shimizu et al. (2006)[31]** | 1 | 1 | 1 | 19 | M | C | IM | Ependymoma | II | Positive | Strong | Not reported | NR |
| **Ewelt et al. (2010)[32]** | 2 | 2 | 1 | 27 | F | T | IM | Anaplastic astrocytoma | III | Positive | Strong | Not reported | GTR |
| **Rapp et al. (2012)[22]** | 3 | 3 | 1 | 31 | M | L | ED | Epidural Metastasis (Anaplastic Oligoastrocytoma) | III | Positive | Strong | Not reported | ST |
|  | 4 | 4 | 2 | 70 | M | T | IM | Drop Metastasis (Glioblastoma) | IV | Positive | Strong | Not reported | NR |
| **Inoue et al. (2013)[13]** | 5 | 5 | 1 | 30 | F | T | IM | Ependymoma | II | Positive | Strong | Uniform | GTR |
|  | 6 | 6 | 2 | 64 | F | C | IM | Ependymoma | II | Positive | Strong | Uniform | GTR |
|  | 7 | 7 | 3 | 37 | M | T | IM | Anaplastic ependymoma | III | Positive | Strong | Non-uniform | GTR |
|  | 8 | 8 | 4 | 25 | F | T | IM | Ependymoma | II | Positive | Strong | Non-uniform | GTR |
|  | 9 | 9 | 5 | 31 | M | C | IM | Ependymoma | II | Positive | Strong | Uniform | GTR |
|  | 10 | 10 | 6 | 39 | M | T | IM | Ependymoma | II | Positive | Strong | Uniform | GTR |
|  | 11 | 11 | 7 | 27 | F | C | IM | Ependymoma | II | Positive | Strong | Non-uniform | GTR |
|  | 12 | 12 | 8 | 53 | F | CT | IM | Ependymoma | II | Negative | N/A | N/A | GTR |
|  | 13 | 13 | 9 | 58 | M | C | IM | Ependymoma | II | Negative | N/A | N/A | ST |
|  | 14 | 14 | 10 | 37 | M | C | IM | Ependymoma | II | N/A | N/A | N/A | ST |
| **Muroi et al. (2013)[30]** | 15 | 15 | 1 | 78 | F | C | ID-EM | Meningothelial meningioma | I | Positive | Not reported | Not reported | S2 |
| **Eicker et al. (2013)[33]** | 16 | 16 | 1 | 67 | F | L | ID-EM | Meningothelial meningioma | I | Positive | Not reported | Not reported | S2 |
|  | 17 | 17 | 2 | 72 | F | T | ID-EM | Psammomatous meningioma | I | Negative | N/A | N/A | S2 |
|  | 18 | 18 | 3 | 47 | F | T | ID-EM | Meningothelial meningioma | I | Positive | Not reported | Not reported | S2 |
|  | 19 | 19 | 4 | 62 | F | C | ID-EM | Meningothelial meningioma | I | Positive | Not reported | Not reported | S2 |
|  | 20 | 20 | 5 | 70 | F | T | ID-EM | Transitional meningioma | I | Positive | Not reported | Not reported | S2 |
|  | 21 | 21 | 6 | 55 | F | T | ID-EM | Transitional meningioma | I | Positive | Not reported | Not reported | S1 |
|  | 22 | 22 | 7 | 82 | F | T | ID-EM | Transitional meningioma | I | Positive | Not reported | Not reported | S2 |
|  | 23 | 23 | 8 | 62 | F | C | ID-EM | Metaplastic meningioma | I | Positive | Not reported | Not reported | S2 |
|  | 24 | 24 | 9 | 39 | M | L | ID-EM | Neurinoma | I | Negative | N/A | N/A | NR |
|  | 25 | 25 | 10 | 71 | M | C | ID-EM | Neurofibroma | I | Negative | N/A | N/A | NR |
|  | 26 | 26 | 11 | 46 | F | T | ID-EM | Neurinoma | I | Negative | N/A | N/A | NR |
|  | 27 | 27 | 12 | 62 | F | S | ID-EM | Neurinoma | I | Negative | N/A | N/A | NR |
|  | 28 | 28 | 13 | 35 | F | L | ID-EM | Neurinoma | I | Negative | N/A | N/A | NR |
|  | 29 | 29 | 14 | 48 | M | C | ID-EM | Neurinoma | I | Negative | N/A | N/A | NR |
|  | 30 | 30 | 15 | 76 | M | L | ID-EM | Neurinoma | I | Negative | N/A | N/A | NR |
|  | 3^a^ | 31 | 16 | 31 | M | L | ED | Epidural metastasis (Anaplastic oligoastrocytoma) | III | Positive | Not reported | Not reported | NR |
|  | 4^a^ | 32 | 17 | 70 | M | T | IM | Drop metastasis (Glioblastoma) | IV | Positive | Not reported | Not reported | NR |
|  | 2^b^ | 32 | 18 | 27 | F | TL | IM | Anaplastic astrocytoma | III | Positive | Not reported | Not reported | NR |
|  | 31 | 34 | 19 | 54 | M | TL | IM | Drop metastasis (Glioblastoma) | IV | Positive | Not reported | Not reported | NR |
|  | 32 | 35 | 20 | 73 | M | TL | ID-EM | Myxopapillary ependymoma | I | Negative | N/A | N/A | NR |
|  | 33 | 36 | 21 | 47 | F | TL | ID-EM | Myxopapillary ependymoma | I | Positive | Not reported | Not reported | NR |
|  | 34 | 37 | 22 | 52 | M | L | ID-EM | Epidermoid cyst | N/A | Negative | N/A | N/A | NR |
|  | 35 | 38 | 23 | 47 | M | T | ID-EM | Drop metastasis (Medulloblastoma) | IV | Negative | N/A | N/A | NR |
|  | 36 | 39 | 24 | 46 | M | S | ID-EM | Drop metastasis (Choroid Plexus Papilloma) | I | Negative | N/A | N/A | NR |
|  | 37 | 40 | 25 | 68 | F | T | IM | Intramedullary lipoma | N/A | Negative | N/A | N/A | NR |
|  | 38 | 41 | 26 | 52 | F | T | IM | Demyelinating disease | N/A | Negative | N/A | N/A | NR |
| **Millesi et al. (2014)[3]** | 39 | 42 | 1 | 58 | M | T | ED | Epidural metastasis (prostate carcinoma) | N/A | Negative | N/A | N/A | B |
|  | 40 | 43 | 2 | 70 | M | T | ED | Epidural metastasis (rectum carcinoma) | N/A | Negative | N/A | N/A | PR |
|  | 41 | 44 | 3 | 44 | F | T | ED | Epidural metastasis (mammary carcinoma) | N/A | Negative | N/A | N/A | B |
|  | 42 | 45 | 4 | 54 | M | L | ED | Epidural metastasis (prostate carcinoma) | N/A | Negative | N/A | N/A | PR |
|  | 43 | 46 | 5 | 38 | M | C | ED | Giant cell tumor of bone | N/A | Negative | N/A | N/A | B |
|  | 44* | 47 | 6 | 22 | M | C | ID-EM | Meningioma NOS | I | Positive | Strong | Uniform | GTR |
|  | 45 | 48 | 7 | 57 | F | TL | ID-EM | Meningothelial meningioma | I | Positive | Strong | Uniform | GTR |
|  | 46 | 49 | 8 | 50 | F | T | ID-EM | Meningothelial meningioma | I | Positive | Strong | Uniform |  |
|  | 47 | 50 | 9 | 57 | F | T | ID-EM | Meningothelial meningioma | I | Positive | Strong | Uniform | GTR |
|  | 48 | 51 | 10 | 66 | M | C | ID-EM | Meningothelial meningioma | I | Positive | Strong | Uniform | GTR |
|  | 49 | 52 | 11 | 81 | F | T | ID-EM | Meningothelial meningioma | I | Positive | Strong | Uniform | GTR |
|  | 50 | 53 | 12 | 56 | F | T | ID-EM | Meningothelial meningioma | I | Positive | Strong | Uniform | GTR |
|  | 51 | 54 | 13 | 73 | F | TL | ID-EM | Meningothelial meningioma | I | Positive | Strong | Non-uniform | GTR |
|  | 52 | 55 | 14 | 61 | F | T | ID-EM | Psammomatous meningioma | I | Positive | Strong | Non-uniform | GTR |
|  | 53 | 56 | 15 | 64 | F | T | ID-EM | Psammomatous meningioma | I | Positive | Strong | Uniform | GTR |
|  | 54 | 57 | 16 | 72 | F | T | ID-EM | Psammomatous meningioma | I | Positive | Strong | Uniform | GTR |
|  | 55 | 58 | 17 | 83 | F | T | ID-EM | Transitional Meningioma | I | Positive | Strong | Uniform | GTR |
|  | 56 | 59 | 18 | 45 | F | T | ID-EM | Neurinoma | I | Negative | N/A | N/A | PR |
|  | 57 | 60 | 19 | 29 | M | C | ID-EM | Neurinoma | I | Negative | N/A | N/A | PR |
|  | 58 | 61 | 20 | 48 | F | T | ID-EM | Neurinoma | I | Negative | N/A | N/A | GTR |
|  | 59 | 62 | 21 | 22 | M | T | ID-EM | Neurinoma | I | Negative | N/A | N/A | GTR |
|  | 60 | 63 | 22 | 22 | M | T | ID-EM | Neurinoma | I | Negative | N/A | N/A | GTR |
|  | 61 | 64 | 23 | 56 | M | C | ID-EM | Neurinoma | I | Negative | N/A | N/A | GTR |
|  | 62 | 65 | 24 | 31 | F | C | ID-EM | Neurinoma | I | Negative | N/A | N/A | GTR |
|  | 63 | 66 | 25 | 51 | F | L | ID-EM | Neurinoma | I | Negative | N/A | N/A | GTR |
|  | 64** | 67 | 26 | 33 | M | T | ID-EM | Hemangiopericytoma | II | Positive | Strong | Uniform | GTR |
|  | 64** | 68 | 27 | 35 | m | C | ID-EM | Hemangiopericytoma | II | Positive | Strong | Uniform | GTR |
|  | 64** | 69 | 28 | 35 | M | C | ID-EM | Anaplastic Hemangiopericytoma | III | Positive | Strong | Uniform | GTR |
|  | 65 | 70 | 29 | 61 | M | L | ID-EM | Chordoma | N/A | Negative | N/A | N/A | PR |
|  | 66 | 71 | 30 | 87 | F | C | ID-EM | Chordoma | N/A | Negative | N/A | N/A | GTR |
|  | 67 | 72 | 31 | 42 | F | T | ID-EM | Drop metastasis (Papillary tumor of Pineal Gland) | II-III | Positive | Strong | Uniform | GTR |
|  | 68 | 73 | 32 | 28 | M | S | ID-EM | Drop metastasis (Glioblastoma) | IV | Positive | Strong | Uniform | B |
|  | 69 | 74 | 33 | 20 | M | L | ID-EM | Myxopapillary ependymoma | I | Positive | Weak | Uniform | GTR |
|  | 70 | 75 | 34 | 69 | F | T | ID-EM | Lipoma | I | Negative | N/A | N/A | PR |
|  | 71 | 76 | 35 | 34 | M | L | ID-EM | Malignant peripheral nerve sheath tumor | II | Negative | N/A | N/A | B |
|  | 72 | 77 | 36 | 28 | M | L | ID-EM | Paraganglioma | I | Negative | N/A | N/A | GTR |
|  | 73 | 78 | 37 | 61 | F | T | IM | Ependymoma | II | Positive | Strong | Non-uniform | ST |
|  | 74 | 79 | 38 | 52 | M | C | IM | Ependymoma | II | Positive | Strong | Non-uniform | GTR |
|  | 75 | 80 | 39 | 48 | F | T | IM | Ependymoma | II | Positive | Strong | Non-uniform | GTR |
|  | 76 | 81 | 40 | 32 | F | T | IM | Ependymoma | II | Positive | Strong | Uniform | GTR |
|  | 44* | 82 | 41 | 22 | M | C | IM | Ependymoma | II | Positive | Strong | Uniform | GTR |
|  | 77 | 83 | 42 | 50 | F | C | IM | Ependymoma | II | Positive | Weak | Non-uniform | GTR |
|  | 78 | 84 | 43 | 49 | M | T | IM | Ependymoma | II | Positive | Strong | Uniform | GTR |
|  | 79 | 85 | 44 | 40 | F | C | IM | Ependymoma | II | Positive | Strong | Uniform | GTR |
|  | 80 | 86 | 45 | 70 | F | CT | IM | Ependymoma | II | Positive | Strong | Non-uniform | ST |
|  | 81 | 87 | 46 | 34 | F | C | IM | Ependymoma | II | Positive | Strong | Non-uniform | ST |
|  | 82 | 88 | 47 | 72 | F | T | IM | Anaplastic ependymoma | III | Positive | Strong | Non-uniform | GTR |
|  | 83 | 89 | 48 | 37 | F | C | IM | Pilocytic astrocytoma | I | Negative | N/A |  | GTR |
|  | 84 | 90 | 49 | 34 | M | T | IM | Diffuse astrocytoma | II | Negative | N/A |  | PR |
|  | 85 | 91 | 50 | 27 | F | TL | IM | Anaplastic oligoastrocytoma | III | Negative | N/A |  | STR |
|  | 86 | 92 | 51 | 45 | M | C | IM | Hemangioblastoma | I | Negative | N/A |  | GTR |
|  | 87 | 93 | 52 | 34 | M | C | IM | Hemangioblastoma | I | Negative | N/A |  | GTR |
|  | 88 | 94 | 53 | 17 | F | C | IM | Ganglioglioma | I | Positive | Strong | Non-uniform | PR |
|  | 89 | 95 | 54 | 42 | F | T | IM | Amelanotic melanocytoma | N/A | Negative | N/A |  | GTR |
|  | 90 | 96 | 55 | 51 | M | T | IM | Intramedullary Metastasis (Squamous cell carcinoma) | N/a | Negative | N/A |  | PR |
| **Kraus Molle et al. (2018)[34]** | 91 | 97 | 1 | 26 | F | L | IM | Anaplastic astrocytoma (leptomenigeal spread) | III | Positive | Weak | Not reported | B |
| *Seg=Segment; C=cervical; T=thoracic; L=lumbar; CT=cervicothoracic junction; TL=thoracolumbar junction*  *Loc=Location; IM=intramedullary; ID-EM=intradural extramedullary; ED=extradural*  *EOR=extent of resection NR=not reported; ST=subtotal resection (macroscopic complete resection with MRI showing residual); PR=partial resection (incomplete removal confirmed by post-operative MRI); GTR=gross total resection; S1=Simpson grade 1 resection; S2=Simpson grade 2 resection; B=biopsy*  *^a^* Previously reported in Rapp et al. (2012) ^b^ Previously reported in Ewelt et al. (2010)  *^*^Patient #44 had a meningioma and intramedullary ependymoma resected during the same operation; ^**^Patient #64 had three hemangiopericytomas resected in two operations.* | | | | | | | | | | | | | |
